# A Pilot Study to Assess the Feasibility of Transcutaneous Glomerular Filtration Rate Measurement Using Fluorescence-Labelled Sinistrin in Dogs and Cats

**DOI:** 10.1371/journal.pone.0111734

**Published:** 2014-11-25

**Authors:** Sarah Steinbach, Nora Krolop, Sellyn Strommer, Zeneida Herrera-Pérez, Stefania Geraci, Jochen Friedemann, Norbert Gretz, Reto Neiger

**Affiliations:** 1 Small Animal Clinic, (Internal Medicine), Justus – Liebig – University, Giessen, Germany; 2 Medical Research Center, University of Heidelberg, Mannheim, Germany; Medical University of Graz, Austria

## Abstract

In dogs and cats an assessment of renal function is often needed, however, existing methods including urine and plasma clearances are invasive, cumbersome and time consuming. This pilot study evaluated the feasibility of a transcutaneous glomerular filtration rate (GFR) measurement in dogs and cats. Additionally the optimal dose and location for the transcutaneous measurement device were investigated. Renal elimination of fluorescein-isothiocyanate-labelled sinistrin (FITC-S) was measured transcutaneously for 4 hours. The procedures were performed in awake, freely moving animals using escalating doses of FITC-S (10 mg/kg, 30 mg/kg, 50 mg/kg) with a wash-out period of at least 24 h in between. Multiple devices were placed on each animal. The resulting FITC-S disappearance curves were visually assessed to determine the most suitable location and the appropriate dose to reach an adequate transcutaneous peak signal for kinetic analysis. In both species 30 mg/kg were adequate for kinetic calculation. The most suitable place for the device was the lateral thoracic wall in dogs and the ventral abdominal wall in cats, respectively. Transcutaneous FITC-S clearance was then repeated using the optimal dose and location and in parallel with an additional plasma sinistrin clearance. Plasma elimination half-lives [min] were 26, 31 and 35, and corresponding transcutaneous elimination half-lives [min] were 26, 34 and 55, respectively in the dogs. Plasma elimination half-lives [min] were 51, 60 and 61, and corresponding transcutaneous elimination half-lives [min] were 75, 96 and 83, respectively in the cats. In conclusion, transcutaneous FITC-S clearance is a feasible method for the assessment of GFR in awake dogs and cats. It is noninvasive, well tolerated and easy to perform even in a clinical setting with results being readily available. A dose of 30 mg/kg of FITC-S seems adequate for kinetic assessment. Further studies are now needed to establish reference values and evaluate transcutaneous renal clearance in various conditions.

## Introduction

Determination of glomerular filtration rate (GFR) by means of plasma or urine clearances of exogenous markers such as inulin (and its equivalent sinistrin), iohexol, radionucleids or creatinine currently is the gold standard in assessing renal function in pets. [1] Urinary clearance procedures are cumbersome and harbor the risk of urinary tract infection owing to catheterization. Hence plasma clearance methods such as the exogenous bolus sinistrin, [2] creatinine, [3] or iohexol clearance, [4] are used in clinical settings. Repeated blood sampling after injection of the respective marker is necessary, although protocols for limited sampling strategies are available. [3], [5], [6] Another approach to determine GFR is to measure the elimination of a fluorescent marker transcutaneously. These procedures showed reliable results in rats and mice and newly developed devices make it even possible to measure GFR in conscious animals. [7]–[11] The transcutaneous approach to measure GFR has not yet been reported in larger animals such as dogs and cats for veterinary use or as model for human patients. A prospective study was designed to test the feasibility of a transcutaneous assessment of renal function using FITC-S kinetics. The aims of the study were threefold: to find the appropriate dose of FITC-S and the optimal position for the optical device in dogs and cats and to see how the transcutaneously measured half-life of FITC-S compares to the half-life obtained by a plasma sinistrin clearance.

## Materials and Methods

### Animals

Three healthy research dogs (female, 2–3 years, 7–9 kg) and three healthy research cats (male, 2 years, 5 kg) were used for the procedures. The study was approved by the state ethics and welfare committee Hessia (number 24/2013). The animals were deemed healthy based on physical exam, complete blood count, biochemistry profile, and urinalysis.

### Experimental design

The study was separated into two parts. First the appropriate location of the devices for transcutaneous measurement as well as the ideal dose of FITC-sinistrin had to be determined. Therefore, the devices for the transcutaneous measurement were placed at four different locations on each animal: antebrachium, metatarsus, lateral thoracic wall, and ventrolateral abdomen. Each animal received escalating doses (10 mg/kg, 30 mg/kg, 50 mg/kg) of FITC-sinistrin at least 24 hours apart. For the FITC-sinistrin solution the commercially available GFR marker Inutest, containing the pharmacological active ingredient sinistrin (Fresenius Kabi, Linz, Austria), was labeled with FITC as described previously [12], [13].

A previously described protocol and device for transcutaneous measurement of FITC-S clearance in rats was used in dogs and cats. [10] The devices (NIC-Kidney; Mannheim Pharma & Diagnostics GmbH, Mannheim, Germany) are built up from 2 light-emitting diodes with an emission maximum of about 480 nm and a photodiode, which detects the light at 520 nm. After amplification and digitalization the data are transferred to a computer program to determine the kinetic parameters.

**Figure 1:**
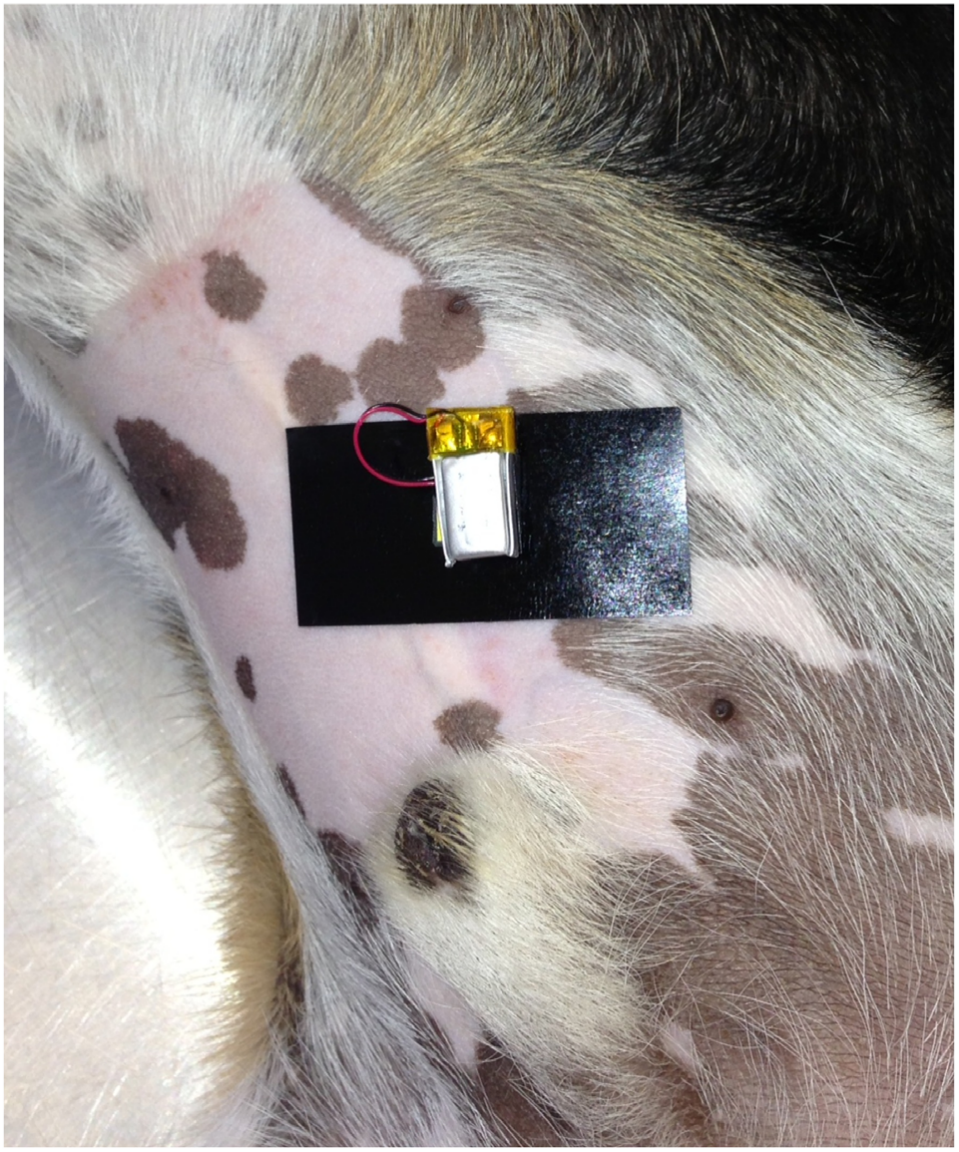
The miniaturized device is placed on a clipped area on the ventral abdomen of a beagle dog and attached using a sticky patch.

An area of 3×3 cm at the chosen locations was shaved and cleaned. For the devices at the antebrachium and metatarsus the leg was circumferentially clipped. The devices were attached to the skin (Fig. 1) using a sticky patch (Lohmann GmbH & Co. KG, Neuwied, Germany) and fixed by a light bandage, insuring that no pressure was applied on the device. After placement of the devices FITC-sinistrin was injected at the respective dose as a bolus via an intravenous catheter and the measurement was continued for 4 hours. During this time the animals were allowed to move freely in an examination room.

The resulting transcutaneous disappearance curves were visually assessed to determine the most suitable location (minimal artifacts, acceptable background noise) and the appropriate dose to reach an adequate transcutaneous peak signal for kinetic analysis. Afterwards the transcutaneous measurement of renal function using the determined ideal dose of FITC-sinistrin and location of the device was performed in parallel with a serum sinistrin clearance as described previously [2], [14].

Briefly, sinistrin was injected as a bolus intravenously immediately after the FITC-sinistrin was administered. To reach a dose of 3000 mg/m^2^ the amount of FITC-sinistrin was subtracted from the total dose and only the remainder injected. Blood samples were taken through a separate venous access at the following time points: 3, 10, 20, 40, 80, 120, and 180 minutes. The exact time points were noted for clearance calculations.

Sinistrin in serum samples as well as the FITC-S solution were analyzed at a commercial laboratory (Alomed, Radolfzell, Germany) as described previously. [2], [14] Analysis of the FITC-S solution was necessary to determine the exact dose of sinistrin administered to allow for kinetic calculations. Plasma sinistrin clearance and plasma half-life were calculated using a 2–compartment model with a freely available pharmacokinetic calculator. [15] Transcutaneous half-life was calculated from the single exponential slope of the transcutaneous disappearance and its rate constant (m) curve via the formula ln(2)/m (one compartment model).

## Results

All procedures were well tolerated in all animals and no side effects were noted.

**Figure 2:**
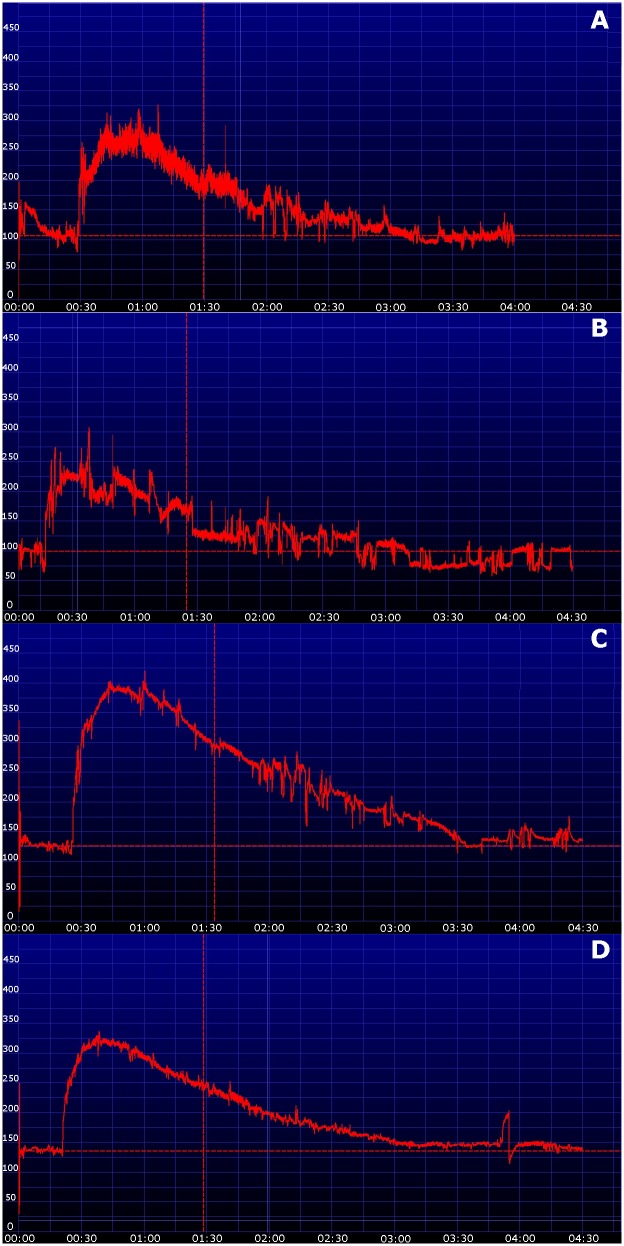
Transcutaneous disappearance curves of fluorescein-isothiocyanate-labelled sinistrin in a dog with the device placed on the four different locations (A – hindlimb, B – forelimb, C – ventral abdomen, D – lateral thorax). The x-axis represents time, the y-axis represents the relative transcutaneous fluorescence. Note that there are minimal artifacts in curve D.

**Figure 3:**
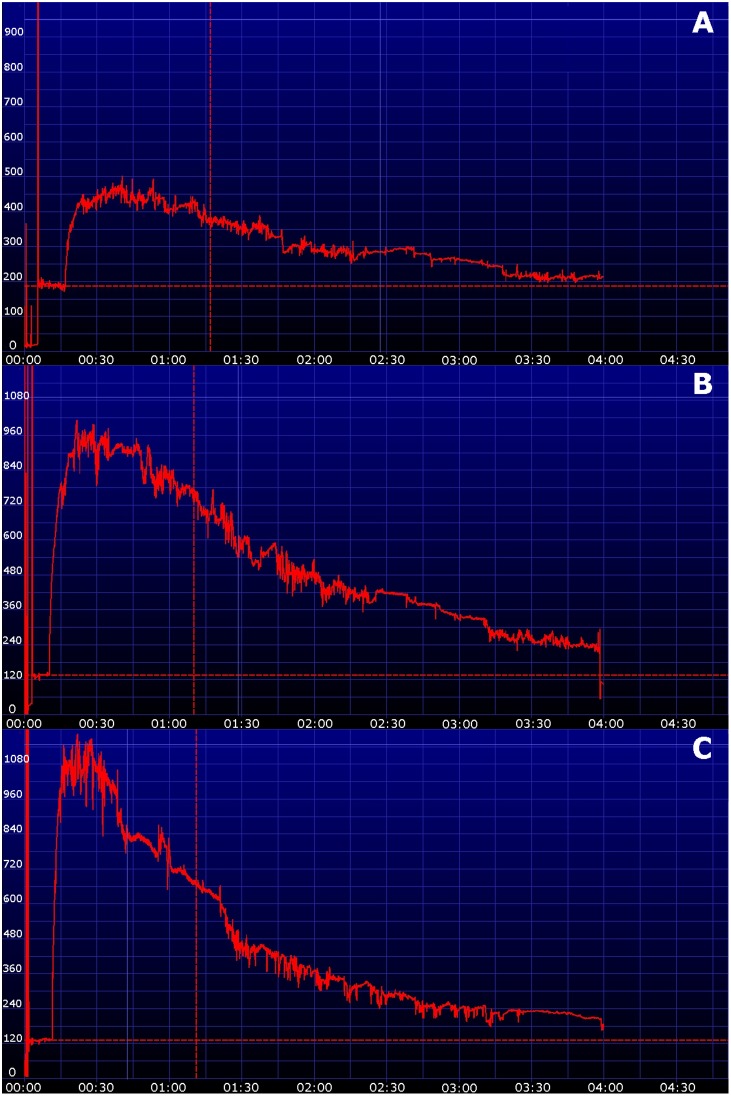
Transcutaneous disappearance curves of a cat using three different dosages of fluorescein-isothiocyanate-labelled sinistrin (A: 10 mg/kg, B: 30 mg/kg, C: 50 mg/kg). The x-axis represents time, the y-axis represents the relative transcutaneous fluorescence. The dose of 30 mg/kg (B) results in an adequate peak concentration.

**Figure 4:**
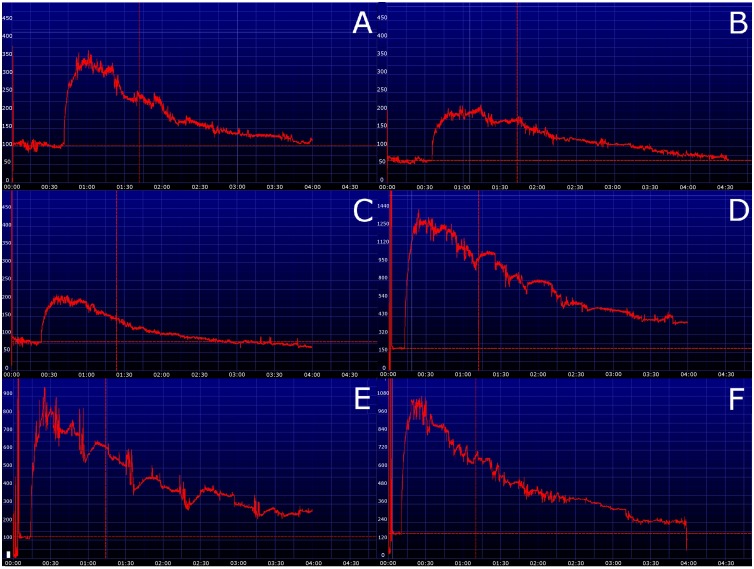
Transcutaneous disappearance curves of fluorescein-isothiocyanate-labelled sinistrin in three dogs (A–C) and three cats (D–F) using a dose of 30 mg/kg.

In dogs the lateral thoracic wall was most appropriate for placement of the devices as there were the least motion artifacts. An example of the different localizations and the resulting transcutaneous disappearance curves in one dog is shown in Fig. 2. In cats the curves obtained at the ventrolateral abdomen and thoracic wall were of equivalent quality. However, instrumentalization (clipping, attachment of the device) was easier to perform at the abdominal wall. Therefore, the subsequent study was performed with the device attached at the ventrolateral abdomen in cats and the thoracic wall in dogs. The dose of 30 mg/kg was deemed adequate based on peak fluorescence and area under the curve in both species. Additionally there was evidence of quenching (extinction of the transcutaneous signal owing to a high optical density from a too high dose of FITC-S) in all three cats using a dose of 50 mg/kg. An example of the resulting transcutaneous disappearance curves using the three different dosages in one cat is shown in Fig. 3. Transcutaneous disappearance curves of all animals using 30 mg/kg FITC-S are shown in Fig. 4. The fitting of the transcutaneous elimination curve was excellent with a r^2^>0.94 in all animals.

Plasma sinistrin data, clearances and half-lives, as well as transcutaneous half-lives are presented in Tables 1 & 2.

**Table 1:** Plasma inulin data in all animals studied.

	Sinistrin (mg/l)					
Time (min)	Dog 1	Dog 2	Dog 3	Cat 1	Cat 2	Cat 3
3	1126	1282	1195	2088	2045	1611
10	540	638	573	1153	1022	920
20	347	416	361	789	732	671
40	191	241	194	573	532	477
80	80	117	74	350	344	313
120	37	64	39	233	228	198
180	19	48	10	127	117	106

**Table 2:** Results of serum sinistrin and transcutaneous fluorescein-isothiocyanate-labelled sinistrin clearance in all animals studied.

Parameter	Dog 1	Dog 2	Dog 3	Cat 1	Cat 2	Cat 3
**Body Weight** (kg)	8.5	9.1	7.0	5.2	4.5	5.0
**Body Surface Area** (m^2^)	0.42	0.44	0.37	0.29	0.27	0.29
**Serum Sinistrin Clearance** (ml/min)	43.7	34.2	38.2	9.9	9.9	13.7
**Serum Sinistrin Clearance** (ml/min/kg)	5.0	3.8	5.5	1.9	2.2	2.7
**Serum Sinistrin Clearance** (ml/min/m^2^)	101.8	77.7	103.1	33.9	36.3	47.1
**Serum Sinistrin half life** (min)	32	35	28	61	60	51
**Transcutaneous FITC-S half life** (min)	34	55	26	83	96	75
**k_10_** (min^−1^)	0.06	0.09	0.08	0.03	0.04	0.03
**k_12_** (min^−1^)	0.08	0.18	0.15	0.09	0.13	0.10
**k_21_** (min^−1^)	0.07	0.07	0.10	0.06	0.07	0.08
**Vss** (ml)	1578	1355	1256	762	755	907

(*FITC-S = fluorescein-isothiocyanate-labelled sinistrin, k = transfer rate between compartments, Vss = Volume of distribution under steady state conditions*).

## Discussion

This study demonstrates that the transcutaneous assessment of FITC-S clearance is feasible in awake dogs and cats. This method has the potential to estimate GFR in these species. It is minimally invasive as only an intravenous injection is needed, but no further blood sampling is necessary. This allows for repeated measurement of GFR even in very small or anemic patients. Another advantage is that the results are readily available.

Our results show that there is considerable variation of half-life when using plasma or transcutaneously derived data. This is especially true for cats and the reason for this is unclear. The normal range or reference interval calculated for different methods of GFR assessment vary considerably. [16] Therefore results obtained by using one method cannot be directly compared to results derived from another method. [1] Establishment of a reference interval using healthy animals with normal renal function and animals with acute and chronic kidney disease is therefore necessary before measuring GFR transcutaneously.

The locations for the placement of the device we found most suitable for analysis are areas which need to be clipped in veterinary medicine for a variety of reasons e.g. abdominal ultrasound, echocardiography, or placement of ECG patches. Therefore no additional clipping is needed.

The determination of half-life or the rate constant *m* as surrogate for renal function is not new in the field of veterinary medicine. Usually GFR is factored to body weight or body surface area. [1] However, standardization to extracellular fluid volume (ECFV) may be a more physiologic approach. The rate constant *m* of the single exponential slope of the disappearance curve is a close estimate of GFR/ECFV in humans and dogs and is converted to half-life via the simple equation ln(2)/*m* [17]–[19].

Determination of GFR/ECFV via plasma clearance of radio-isotopes and by an external radiation detector has been previously performed in dogs. However, considering radiation safety, this technique is not widely used. [19] Unfortunately the authors do not report absolute values for *m* making a direct comparison of the two studies impossible. Although one has to consider that the use of a one compartment model for estimating GFR is not ideal and might overestimate renal clearance especially in individuals with normal renal function [1], [20], it is the only method which can be used when assessing transcutaneously derived data [9].

To compare the transcutaneously measured clearance with the existing literature or if the conventional indexing to body surface area or body weight is required, clearance can be calculated from half-life using a species specific conversion factor as it has been shown for rats. [9] As this was a study of feasibility, only a small number of animals were included, rendering the calculation of such a formula impossible.

The transcutaneous device is lightweight and all animals tolerated the measurement well. Especially for cats this is a very attractive way of assessing GFR as restriction is only needed to inject the marker substance intravenously and fix the device. No further manipulation is necessary. Moreover, results of the GFR measurement are readily available to the clinician.

Skin blood flow is independent of skin thickness, however, both parameters vary between species. Additionally, these parameters vary in different body areas within an animal. [21] Therefore caution is warranted if comparisons are drawn in future studies as only data from the same location in the same species should be compared. Furthermore, the use on pigmented or inflamed skin may lead to decreased or increased signal amplitude, respectively. Further studies are necessary to evaluate how skin condition and pigmentation may influence the transcutaneous measurement of FITC-S.

In conclusion, transcutaneous FITC-S clearance is a feasible and non-invasive method for assessment of GFR in awake dogs and cats. Further studies are necessary to establish reference values for the transcutaneous half-life of FITC-S in healthy animals and animals with varying degree of renal disease.
